# TGFβ isoforms and receptors mRNA expression in breast tumours: prognostic value and clinical implications

**DOI:** 10.1186/s12885-015-1993-3

**Published:** 2015-12-24

**Authors:** Chenfeng Chen, Kong-Nan Zhao, Paul P. Masci, Sunil R. Lakhani, Annika Antonsson, Peter T. Simpson, Luis Vitetta

**Affiliations:** 1 The University of Queensland, School of Medicine, Level 5, Translational Research Institute, 37 Kent Street, Woolloongabba, Brisbane, QLD 4102 Australia; 2 The University of Queensland, UQ Centre for Clinical Research, Brisbane, Australia; 3 Pathology Queensland, The Royal Brisbane & Women’s Hospital, Brisbane, Australia; 4 QIMR Berghofer Medical Research Institute, Brisbane, Australia; 5 Medlab Clinical, Sydney, Australia; 6 The University of Sydney, Sydney Medical School, Sydney, Australia

**Keywords:** TGFβ, Breast cancer, mRNA expression, Prognosis

## Abstract

**Background:**

Transforming growth factor beta (TGFβ) signalling is involved in both tumour suppression and tumour progression. The mRNA expression levels of the TGFβ isoforms and receptors in breast tumours may have prognostic value and clinical implications.

**Methods:**

The mRNA levels of *TGFB1*, *TGFB2*, *TGFB3*, *TGFBR1* and *TGFBR2* were analysed in primary breast tumours and adjacent normal breast tissues, and the associations with tumour characteristics and patients’ overall and relapse-free survival were evaluated, using the public gene expression microarray data from The Cancer Genome Atlas (*n* = 520) and the Gene Expression Omnibus (four datasets) and our quantitative real-time PCR validation data (*n* = 71).

**Results:**

Significantly higher *TGFB1* and *TGFB3* mRNA levels and lower *TGFBR2* mRNA levels were observed in primary tumours compared with their paired normal tissues. *TGFB1* mRNA expression was seemly lower in triple-negative tumours and in tumours from lymph node-negative patients. *TGFB3* mRNA expression was significantly lower in estrogen receptor-negative/progesterone receptor-negative/Basal-like/Grade 3 tumours. High *TGFB2*, *TGFB3* and *TGFBR2* mRNA levels in tumours were generally associated with better prognosis for patients, especially those diagnosed with lymph node-negative diseases. High *TGFBR1* mRNA levels in tumours were associated with poorer clinical outcomes for patients diagnosed with small (diameter ≤2 cm) tumours.

**Conclusions:**

The results indicate a reduced responsiveness of tumour cells to TGFβ, a preferential up-regulation of *TGFB1* in malignant tumours and a preferential up-regulation of *TGFB3* in premalignant tumours. The results may not only provide prognostic value for patients but also assist in classifying tumours according to their potential responses to TGFβ and selecting patients for TGFβ signalling pathway targeted therapies.

## Background

Transforming growth factor beta (TGFβ) signalling is involved in the maintenance of tissue homeostasis and suppression of premalignant tumour cells, however, when the regulations are circumvented, TGFβ signalling can be advantageously exploited by the tumour cells to promote tumour progression and metastasis [1]. Three isoforms of TGFβ (TGFβ1, TGFβ2 and TGFβ3) all function as secreted polypeptides that can modulate the cellular microenvironment [2]. When activated, TGFβ binds to and brings together transmembrane TGFβ receptors type I (TβRI) and type II (TβRII) to form a ligand-receptor complex that propagates the signal to the nucleus [3]. The effects and mode of action of TGFβ are varied and depend on the cellular context [4].

Extensive efforts have been made to investigate the role of TGFβ signalling in the development and progression of cancer and to develop drugs targeting the TGFβ signalling pathway for a number of cancer types, including breast cancer [1, 5–7]. The prognostic value of the components of TGFβ pathway have also been explored in breast cancer, though conflicting results have been noted [8–11]. Much of our knowledge about TGFβ signalling in breast cancer is based on the studies characterizing the proteins involved. A few pioneer studies have evaluated the mRNA levels [12, 13], whereas whether the mRNA levels of the key components of the TGFβ pathway also have prognostic value and clinical implications is inconclusive.

Here, we used the public breast cancer gene expression microarray datasets from The Cancer Genome Atlas (TCGA) and the Gene Expression Omnibus (GEO) to compare the mRNA expression levels of *TGFB1*, *TGFB2*, *TGFB3*, *TGFBR1* and *TGFBR2* in primary tumours and tumour-adjacent normal tissues, and to evaluate the associations between the mRNA levels and the clinical, pathological and molecular tumour characteristics and the patients’ overall and relapse-free survival. We also validated the gene expression profiles on an independent set of breast cancer surgical specimens by reverse-transcription quantitative real-time polymerase chain reaction (RT-qPCR).

## Methods

### Study population and characteristics of breast cancer specimens

The TCGA breast cancer level 3 mRNA expression Agilent microarray data (Lowess normalized and log2-transformed) and the patients’ clinical data were downloaded from the Broad Genome Data Analysis Centre Firehose (April 2015 version). The TCGA cohort (Table 1) involved 520 untreated primary breast tumour samples and 59 paired tumour-adjacent normal tissue samples (taken from greater than 2.0 cm away from the tumour) from 520 females (age range 26–90 years). The bio-specimen collection criteria, sample processing, clinical data quality assurance and microarray processing had been described by TCGA previously [14]. The pathologic stages, namely, primary tumour (T), regional lymph nodes (N) and distant metastases (M) were made binary in this study: tumour size was coded as “≤2 cm” for T1, “>2 cm” for T2 and T3, and “Not Available” (NA) for T4 and TX; involvement of regional lymph nodes was coded as “negative” for N0, NA for NX and “positive” for the others; distant metastasis was coded as "positive" for M1 and "negative" for the others. Immunohistochemical data was used for determining estrogen receptor (ER), progesterone receptor (PR) and human epidermal growth factor receptor 2 (HER2) statuses. HER2 status was supplemented by the results of fluorescence in situ hybridization for the “equivocal” or NA calls. Scores of “indeterminate”, “equivocal” or “not performed” were coded as NA. PAM50 subtypes for 513 of the tumour samples had been previously assigned [14], and that for the other seven tumour samples were predicted using the 50-gene PAM50 model [15].

**Table 1:** Tumour characteristics of the breast cancer specimens

Cohorts	TCGA	TCGA-sub	RT-qPCR	Ivshina	Schmidt	Symmans	Wang
N	520	59	71	249	200	298	286
Histological type							
Ductal	442 (85.0)	51 (86.4)	56 (78.9)	–	–	–	–
Lobular	41 (7.9)	3 (5.1)	5 (7.0)	–	–	–	–
Mixed and others	36 (6.9)	5 (8.5)	9 (12.7)	–	–	–	–
Tumour diameter							
≤2.0 cm	133 (25.6)	20 (33.9)	39 (54.9)	126 (50.6)	112 (56.0)	–	–
>2.0 cm	364 (70.0)	38 (64.4)	32 (45.1)	123 (49.4)	88 (44.0)	–	–
Regional lymph nodes							
Negative	253 (48.7)	25 (42.4)	34 (47.9)	159 (63.9)	200 (100)	175 (58.7)	286 (100)
Positive	256 (49.2)	34 (57.6)	36 (50.7)	81 (32.5)	0 (0)	112 (37.6)	0 (0)
Distant metastasis							
Negative	491 (94.4)	57 (96.6)	–	–	–	–	–
Positive	15 (2.9)	1 (1.7)	–	–	–	–	–
TNM stage							
I	89 (17.1)	13 (22.0)	–	–	–	–	–
II	290 (55.8)	34 (57.6)	–	–	–	–	–
III	110 (21.2)	11 (18.6)	–	–	–	–	–
IV	14 (2.7)	1 (1.7)	–	–	–	–	–
Tumour grade							
Grade 1	–	–	7 (9.9)	68 (27.3)	29 (14.5)	–	–
Grade 2	–	–	25 (35.2)	126 (50.6)	136 (68.0)	–	–
Grade 3	–	–	36 (50.7)	55 (22.1)	35 (17.5)	–	–
ER status							
Negative	117 (22.5)	9 (15.3)	18 (25.4)	34 (13.7)	–	0 (0)	77 (26.9)
Positive	396 (76.2)	48 (81.4)	52 (73.2)	211 (84.7)	–	298 (100)	209 (73.1)
PR status							
Negative	176 (33.8)	16 (27.1)	22 (31.0)	–	–	–	–
Positive	336 (64.6)	41 (69.5)	48 (67.6)	–	–	–	–
HER2 status							
Negative	361 (69.4)	48 (81.4)	60 (84.5)	–	–	–	–
Positive	101 (19.4)	10 (16.9)	10 (14.1)	–	–	–	–
Triple-negative							
True	72 (13.8)	7 (11.9)	11 (15.5)	–	–	–	–
False	448 (86.2)	52 (89.1)	60 (84.5)	–	–	–	–
PAM50 subtype							
Basal-like	96 (18.5)	11 (18.6)	–	–	–	–	–
HER2-enriched	58 (11.2)	4 (6.8)	–	–	–	–	–
Luminal A	231 (44.4)	32 (54.2)	–	–	–	–	–
Luminal B	127 (24.4)	12 (20.3)	–	–	–	–	–
Normal-like	8 (1.5)	0 (0)	–	–	–	–	–

Data are presented as the number of patients in each subgroup with percentage in the parentheses. Numbers may not sum to total (N) due to missing datadata

A total of four breast cancer microarray datasets from GEO were included in this study (Table 1). The selection criteria were that the dataset should contain ≥200 clinical specimens and there was a published paper associated with it. The Ivshina cohort (GSE4922) was of unselected population [16]. The Schmidt (GSE11121) and Wang (GSE2034) cohorts were both composed of the tumour specimens from lymph node-negative patients [17, 18]. The Symmans cohort (GSE17705) was composed of ER-positive tumour specimens [19].

The RT-qPCR validation cohort was composed of 71 tumour surgical specimens (containing >50 % tumour cells) from 71 females (age range 33–85 years) diagnosed with breast cancer (Table 1). Written informed consent was obtained from all patients. Ethical approvals were obtained from the Human Research Ethics Committee at the University of Queensland (UQ) and Royal Brisbane & Women’s Hospital before the study was conducted. The tumour specimens were snap frozen after surgery and stored in liquid nitrogen at UQ Centre for Clinical Research (the Brisbane Breast Bank) and Wesley hospital, Brisbane, until required for RNA extraction.

### Processing of GEO datasets

The gene expression data of the four GEO datasets were all based on Affymetrix U133A and U133A&B array sets. The data of the probe sets 203084_at (*TGFB1*), 220406_at (*TGFB2*), 209747_at (*TGFB3*), 206943_at (*TGFBR1*) and 208944_at (*TGFBR2*) from each GEO dataset and each subset of the Ivshina dataset (stratified by tumour size, presence or absence of regional lymph nodes and ER status) were log2-transformed and standardized to mean = 0 and standard deviation (SD) = 1. The Ivshina cohort was renamed as “GEO cohort, All”, which was used to represent unselected population. The log2-transformed and standardized gene expression data of the Schmidt cohort, the Wang cohort and the lymph node-negative subset of the Ivshina cohort were merged to form “GEO cohort, N neg.”; and the log2-transformed and standardized gene expression data of the Symmans cohort and the ER-positive subset of the Ivshina cohort were merged to form “GEO cohort, ER pos.”. The T ≤2 cm, T >2 cm, lymph node-positive and ER-negative subsets of the Ivshina cohort were named as “GEO cohort, T ≤2 cm” “GEO cohort, T >2 cm”, “GEO cohort, N pos.” and “GEO cohort, ER neg.”, respectively. The Ivshina dataset contained recurrence-free survival data (local, regional or distant), while the other three datasets contained distant relapse-free survival data. Thus the merged survival data was referred as relapse-free survival data but with bias to more distant relapse-free survival data.

### Reverse-transcription quantitative real-time polymerase chain reaction

Total RNA was extracted from each specimen using TRIzol reagent (Life Technologies), according to the manufacturer’s instructions. RNA concentration, integrity and purity were analysed using NanoDrop 1000 Spectrophotometer (Thermo Scientific) and agarose gel electrophoresis. Quality-checked 500 ng RNA per specimen was reverse transcribed using high capacity RNA-to-cDNA kit (Life Technologies) in a final reaction volume of 12 μL containing random hexamer primers. The incubation conditions for reverse transcription (RT) were: 37 °C for 60 min, 95 °C for 10 min and hold at 4 °C indefinitely. RT-negative controls (reactions with no reverse transcriptase) of two RNA samples were prepared for testing DNA contamination. The cDNA was kept at −20 °C until used for quantitative real-time PCR (qPCR).

The qPCR was performed using SYBR Green PCR master mix (Life Technologies) in MicroAmp optical 384-well reaction plates (Life Technologies) on an Applied Biosystems 7900HT Fast Real-Time PCR System. The primers for qPCR were designed using AlleleID 7.6 (PREMIER Biosoft) to span at least one exon-exon junction and to cover all the transcript variants found in Entrez Gene (Table 2). Each qPCR reaction had a total volume of 10 μL that contained 12.5 ng cDNA templates, 200 nM forward primers, 200 nM reverse primers and 5 μL SYBR Green PCR master mix. The thermal cycling conditions were: 95 °C for 10 min, followed by 40 cycles of 95 °C for 15 s and 60 °C for 1 min, and a dissociation stage. The qPCR reactions containing the same cDNA template with the whole set of qPCR primer pairs were run on the same plate to avoid assay variability. Every combination of cDNA template and qPCR primer pairs was tested in triplicate. Each qPCR product was verified by dissociation curve analysis, and all the qPCR products of two cDNA templates were verified by 2.2 % agarose gel electrophoresis that showed single band with correct amplicon length and no primer dimmer. There was no positive amplification observed in the qPCR reactions using the RT-negative controls as template. The threshold cycle (Ct) was determined by setting the threshold to 0.1, in the exponential phase of amplification, in the SDS 2.4 software (Life Technologies). Triplicates with median Ct ≤30 all had SD <0.1, while those with median Ct >30 had relatively increased SD, due to obvious higher variability in templates with low transcript copy-number. The median Ct for each triplicate was obtained for further calculations. The qPCR efficiencies of all studied genes were similar and close to 100 %, i.e. 1 thermal cycle (expressed as Ct) corresponds to a 2-fold change.

**Table 2:** Characteristics of the primers used for RT-qPCR

Gene symbol	Targeted transcript variants	Primer sequences (5′ – 3′)	Amplicon (bp)
*TGFB1*	NM_000660	(F): TCGCCAGAGTGGTTATCTT	148
		(R): TAGTGAACCCGTTGATGTCC	
*TGFB2*	NM_001135599, NM_003238	(F): ACACTCAGCACAGCAGGGTCCT	80
		(R): TTGGGACACGCAGCAAGGAGAAG	
*TGFB3*	NM_003239	(F): TGAGTGGCTGTTGAGAAGAGA	139
		(R): ATTGTCCACGCCTTTGAATTTGAT	
*TGFBR1*	NM_001130916, NM_004612	(F): GCAGAGCTGTGAAGCCTTGAGA	128
		(R): TGCCTTCCTGTTGACTGAGTTG	
*TGFBR2*	NM_001024847, NM_003242	(F): ATGACATCTCGCTGTAATGC	163
		(R): GGATGCCCTGGTGGTTGA	
*HPRT1*	NM_000194	(F): TGGCGTCGTGATTAGTGATG	160
		(R): GCCTCCCATCTCCTTCATC	
*GAPDH*	NM_001256799, NM_002046	(F): TCTCTGCTCCTCCTGTTC	112
		(R): CGACCAAATCCGTTGACT	

*F* forward primer, *R* reverse primer, *bp* base pair

The 2^-∆∆Ct^ method was used for the qPCR data analysis [20]. *GAPDH* and *HPRT1* were chosen as the reference genes for normalization, as they showed smaller SD of Ct values compared with two other putative housekeeping genes *RNA18S5* and *GUSB* that were assessed in our pilot study. The ∆Ct for the gene of interest was calculated by subtracting the average of Ct for *GAPDH* and *HPRT1* from the Ct for the gene of interest [21]. The relative mRNA expression levels of the genes of interest (2^-∆Ct^) were skewed, thus the normalized and log2-transformed data, i.e. -∆Ct, was used for data analysis [20].

### Statistical analysis

Log2-transformed and normalized mRNA expression data was used for all statistical analyses. The Wilcoxon signed-rank test was used to test for differences in the mRNA levels between tumours and matched normal tissues. The Spearman’s rank correlation analysis was employed to evaluate the correlations between the mRNA levels of pairwise genes. The Wilcoxon rank-sum test and Kruskal-Wallis test were employed to test for differences in the mRNA levels in subgroups stratified by tumour characteristics, namely, tumour diameter, regional lymph nodes involvement, distant metastasis, ER status, PR status, HER2 status, triple-negative, histological type, TNM stage, tumour grade and PAM50 subtype.

The Cox proportional hazards regression model was used to evaluate the associations between tumour levels of *TGFB1*, *TGFB2*, *TGFB3*, *TGFBR1* and *TGFBR2* mRNA and patients’ overall and relapse-free survival in the TCGA and GEO cohorts. Patient’s survival time was calculated from the date of diagnosis to the date of event (death or relapse) or censored at the last follow-up or last known alive time whichever was longer. To evaluate if the tumour mRNA levels had different associations with patients’ clinical outcomes in the early and late years after patients were diagnosed with breast cancer, the Cox analyses were performed at different periods of follow-up time from 1 to 10 years. For each Cox model, the proportional hazards assumption was evaluated, and the results indicated that some of the gene expression variables were time-dependent. Thus extended Cox models using a Heaviside function approach [22] were applied to calculate the hazard ratios for the period for 0 to less than 3 years and the open-ended period from 3 years and beyond.

To evaluate if stratifying patients by the tumour mRNA levels would predict patients’ survival, the patients of each cohort were divided into two groups according to their tumour mRNA levels of each gene, and then the relevant hazard ratios for different periods of follow-up time (from 1 to 10 years) were calculated using Cox proportional hazards regression model. To avoid arbitrary grouping, the cut point was set to include 10–90 % of the patients with the lowest expression in each cohort. Representative Kaplan-Meier curves were plotted for the cut points that produced the lowest Wald test p-values for HR. The Likelihood-ratio test was used to assess statistical significance of the calculated Cox regression coefficients (β) and hazard ratios (HR), where β = ln(HR). The 95 % confidence interval (CI) of HR was calculated based on the Wald test. The Log-rank test was used to assess significant differences between the Kaplan-Meier estimates.

All the statistical analyses were performed using R (version 3.2.0, R Core Team, 2015). The following R packages were used: qvalue, GEOquery, rms, heatmap.plus, ggplot2, gplots, gridExtra and reshapes2. All the p-values (P) presented were two-sided, and the significance level was set at 0.05. Bonferroni correction for multiple testing was applied where appropriate and corrected p-values were given the symbol P’. For comparison, Storey’s false discovery rate (FDR) procedure, a less stringent multiple testing correction method, was also applied where appropriate [23].

## Results

The bio-specimens from the TCGA, GEO and RT-qPCR cohorts were all collected from newly diagnosed patients with breast tumours that had received no prior treatment, such as chemotherapy or radiotherapy. The clinical, pathological and molecular characteristics of the breast cancer specimens were summarised in Table 1. The population of tumours from the TCGA cohort was not significantly different from the TCGA sub-cohort that contained adjacent normal tissues and the RT-qPCR cohort in terms of the tumour characteristics, with the only exception being “tumour diameter” (Fisher’s exact test *P* = 3.6 × 10^−6^), in which the tumours from TCGA were overall larger than the tumours from the local tissue banks.

### Comparison of mRNA levels between primary breast tumours and adjacent normal tissues

**Fig. 1:**
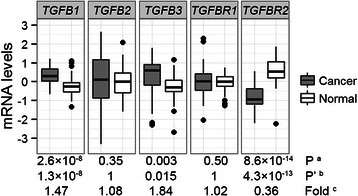
Comparison of mRNA levels between primary breast tumours and adjacent normal tissues. The mRNA expression levels (log2-transformed and median-centred) of *TGFB1*, *TGFB2*, *TGFB3*, *TGFBR1* and *TGFBR2* were compared between 59 primary breast tumours and their adjacent normal tissues from the TCGA cohort using ^a^ the Wilcoxon signed-rank test. ^b^ Bonferroni corrected p-values. ^c^ The relative median mRNA expression levels in cancer versus normal tissues

Significantly higher *TGFB1* and *TGFB3* mRNA levels and lower *TGFBR2* mRNA levels were observed in the primary breast tumours compared with their matched normal tissues (Fig. 1). Higher variance of *TGFB2* mRNA levels was observed compared with the other genes. TGFβ1 and TGFβ3 are generally considered more alike than either is compared to TGFβ2 in term of functions, given that TGFβ1 and TGFβ3 are capable of binding directly to TβRII, while TGFβ2 binds TβRII weakly and is dependent upon the co-receptor betaglycan for function [24]. Interestingly, positive correlations between the mRNA levels of *TGFBR2* and the mRNA levels of *TGFB1* (Spearman’s rho = 0.51, *P* = 5.6 × 10^−5^, P’ = 5.6 × 10^−4^) and *TGFB3* (Spearman’s rho = 0.42, *P* = 0.0012, P’ = 0.012) were observed in the tumours (*n* = 59).

### Associations between tumour mRNA levels and tumour characteristics

**Fig. 2:**
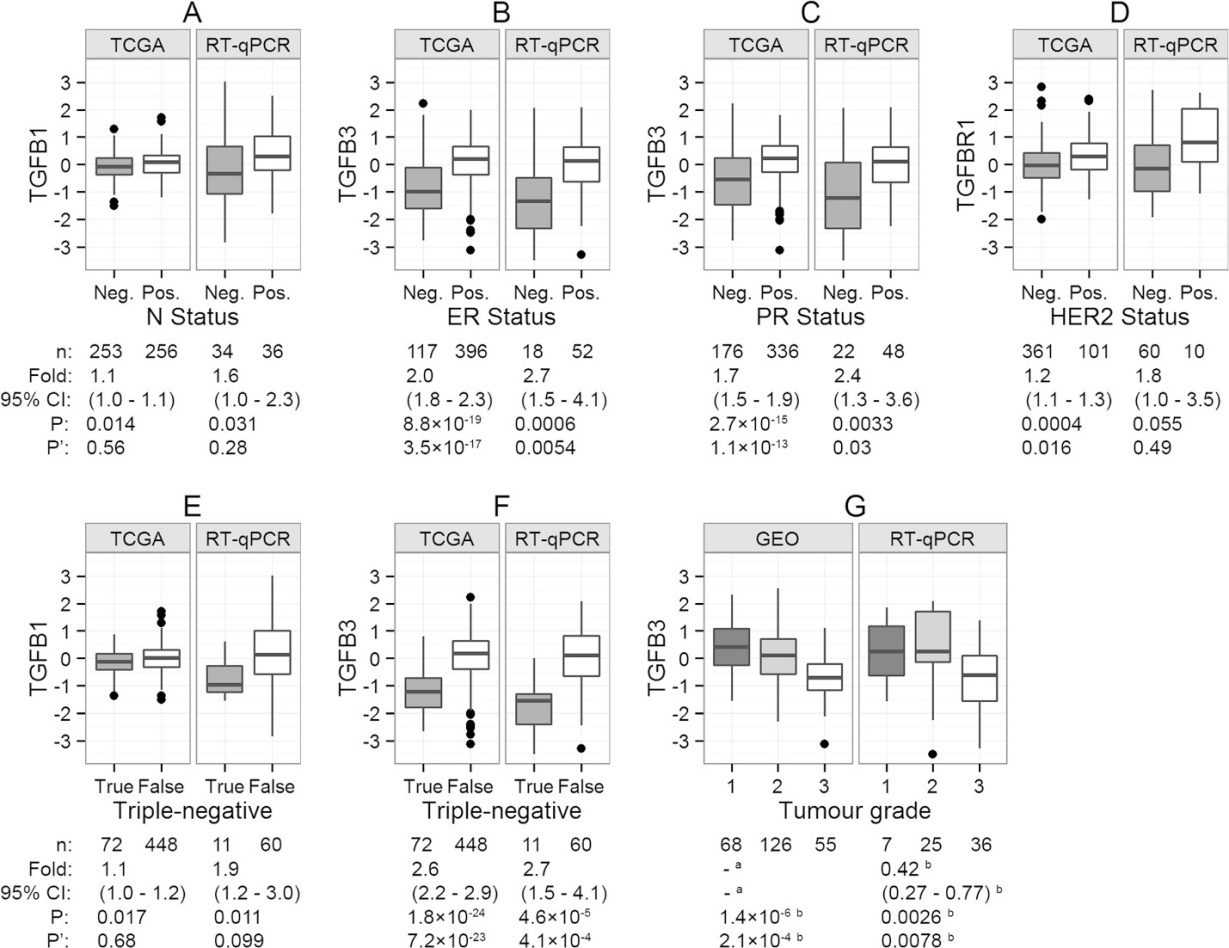
Associations between tumour mRNA levels and tumour characteristics. The mRNA expression levels (log2-transformed and median-centred) of *TGFB1*, *TGFB2*, *TGFB3*, *TGFBR1* and *TGFBR2* (labels on y-axis) were compared between different subgroups of breast tumours (grouped by tumour characteristics, labels on x-axis) from different cohorts (titles on each panel). The analysis were initially performed in the TCGA cohort or in the GEO cohort, and the tests with p-values <0.05 were verified in the RT-qPCR cohort. Comparison of tumour *TGFB1* mRNA levels by (**a**) regional lymph node status and (**e**) triple-negative status. Comparison of tumour *TGFB3* mRNA levels by (**b**) ER status, (**c**) PR status, (**f**) triple-negative status and (**g**) tumour grade. Comparison of tumour *TGFBR1* mRNA levels by (**d**) HER2 status. The number of patients (n) of each tumour subgroup was shown under each boxplot. The estimated relative mRNA expression levels (Fold) in the tumour subgroup on the right side in each panel (Positive, False or grade 3, presented as white boxplot) versus the one on the left side (Negative, True or grade 2, presented as grey boxplot), 95 % confidence intervals and p-values were calculated from the Wilcoxon rank-sum test. ^a^ The data presented were standardized, thus no fold change was calculated. ^b^ The relevant values were calculated for grade 2 and grade 3 tumours

The associations between the mRNA levels of *TGFB1*, *TGFB2*, *TGFB3*, *TGFBR1* and *TGFBR2* and the tumour characteristics displayed in Table 1 were evaluated using Wilcoxon rank-sum test and Kruskal-Wallis test. The significant associations that were observed from both of the TCGA and RT-qPCR cohorts were shown in Fig. 2. The mRNA levels of *TGFB3* was significantly lower in the ER-negative, PR-negative and triple-negative tumours (Fig. 2b, c and f). It was also shown that *TGFB3* mRNA had significantly the lowest expression in the Grade 3 tumours (Fig. 2g), which agreed with a few studies evaluating the mRNA and protein levels of *TGFB3* [25, 26]. Seemly lower *TGFB1* mRNA levels were observed in the tumours from regional lymph node-negative patients and in the triple-negative tumours, and lower *TGFBR1* mRNA levels were observed in HER2 negative tumours (Fig. 2a, d and e), though the corresponding *p*-values did not survive Bonferroni correction.

**Fig. 3:**
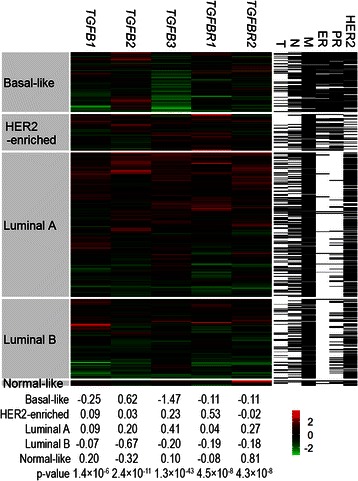
Heatmap visualization of tumour mRNA levels. Tumour samples from the TCGA cohort were hierarchically clustered according to the log2-transformed and standardized mRNA expression levels of *TGFB1*, *TGFB2*, *TGFB3*, *TGFBR1* and *TGFBR2* for each PAM50 subtype in the middle panel, where each row represented a tumour specimen and each column represented a gene. The colour represented the mRNA expression levels (*green*: low, *red*: high). The median mRNA levels of each gene for each PAM50 subtype were shown at the bottom, and the p-values were calculated from the Kruskal-Wallis test for differences in the mRNA levels across the PAM50 subtypes. All the p-values were still significant after Bonferroni correction for multiple testing. The tumour diameter (T), lymph node metastasis (N), distant metastasis (M), ER PR and HER2 statuses of each specimen were shown in the right-side columns, white: positive or >2 cm, black: negative or ≤2 cm, *grey*: missing data

All the five genes were expressed to variable levels across different PAM50 subtypes, and exhibited heterogeneous expression profiles within each PAM50 subtype (Fig. 3). For example, in the Luminal A subtype, some tumours had high expression of all five genes while some had low expression of all five genes. Significantly lower expression of *TGFB3* was observed in the Basal-like tumours, which was in agreement with that the lower expression of *TGFB3* was associated with ER, PR-negative tumours and that most of the Basal-like tumours were ER, PR-negative (Fig. 3). *TGFBR2* had the highest median expression levels in the Normal-like subtype (Fig. 3), which was in concordance with the result that *TGFBR2* was significantly down-regulated in the tumours compared with their adjacent normal tissues (Fig. 1).

### Associations between tumour mRNA levels and patients’ clinical outcomes

**Fig. 4:**
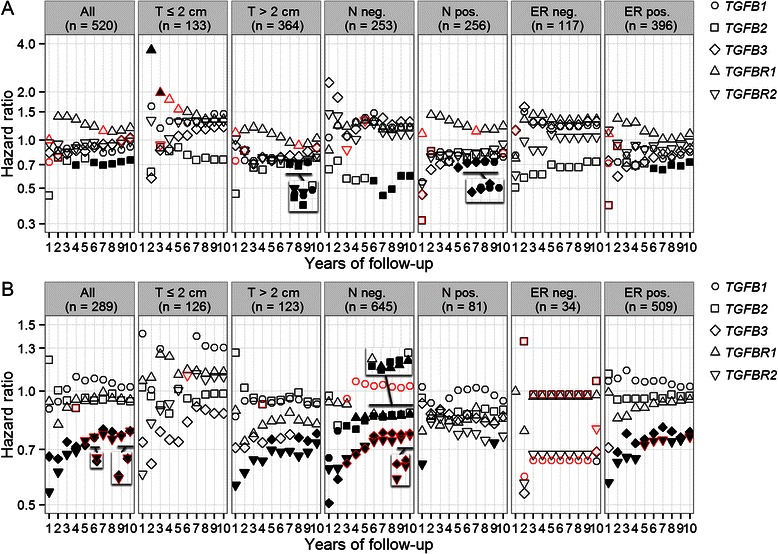
Associations between tumour mRNA levels and patients’ clinical outcomes. **a** The associations between primary breast tumour mRNA levels of *TGFB1*, *TGFB2*, *TGFB3*, *TGFBR1* and *TGFBR2* and patients’ overall survival were evaluated in the TCGA cohort and its subsets stratified by tumour diameter (T), regional lymph node status (N) and ER status using the Cox proportional hazards regression model. **b** Associations between primary breast tumour mRNA levels and patients’ relapse-free survival in the GEO cohorts. The hazard ratios of death (y-axis, logarithmic scale) for a 1-unit increase in the mRNA expression levels (log2-transformed and standardized) of the five genes for the follow-up periods from 1 to 10 years (x-axis) were shown for each group of patients (title on each panel). The shapes were filled black if the corresponding p-values for likelihood-ratio test were <0.05. The border of shape was coloured red if the corresponding proportional hazards assumption was not met (*P* <0.1). Missing data in the first column of panel “T ≤2 cm” in panel A were due to 0 event recorded for the first year. The inserts are the same plots as the underscored ones but having zoomed in y-axis to show the overlays

**Fig. 5:**
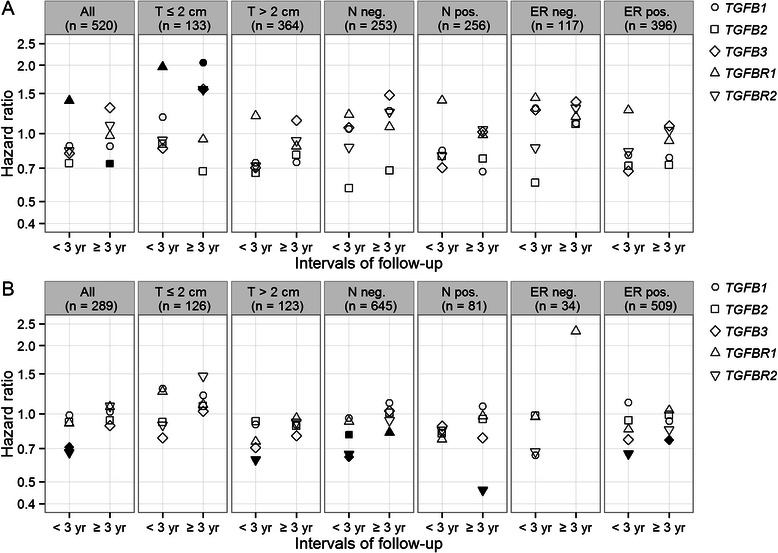
Time-dependent Cox regression analysis for associations between tumour mRNA levels and patients’ clinical outcomes. **a** Time-dependent Cox regression analysis for the associations between primary breast tumour mRNA levels of *TGFB1*, *TGFB2*, *TGFB3*, *TGFBR1* and *TGFBR2* and patients’ overall survival in the TCGA cohort and its subsets stratified by tumour diameter (T), regional lymph node status (N) and ER status. **b** Associations between primary breast tumour mRNA levels and patients’ relapse-free survival in the GEO cohorts. A Heaviside function approach were applied to calculate the hazard ratios of death (y-axis, logarithmic scale) for a 1-unit increase in the mRNA expression levels (log2-transformed and standardized) of the five genes for the period for 0 to less than 3 years and the open-ended period from 3 years and beyond, for each group of patients (title on each panel). The proportional hazards assumption was met (*P* ≥0.1) for each of the separate time intervals <3 years and ≥3 years. The shapes were filled black if the corresponding p-values for likelihood-ratio test were <0.05. Missing data in the panel “ER neg.” were due to exceeding the y-axis limit and were non-significant

**Fig. 6:**
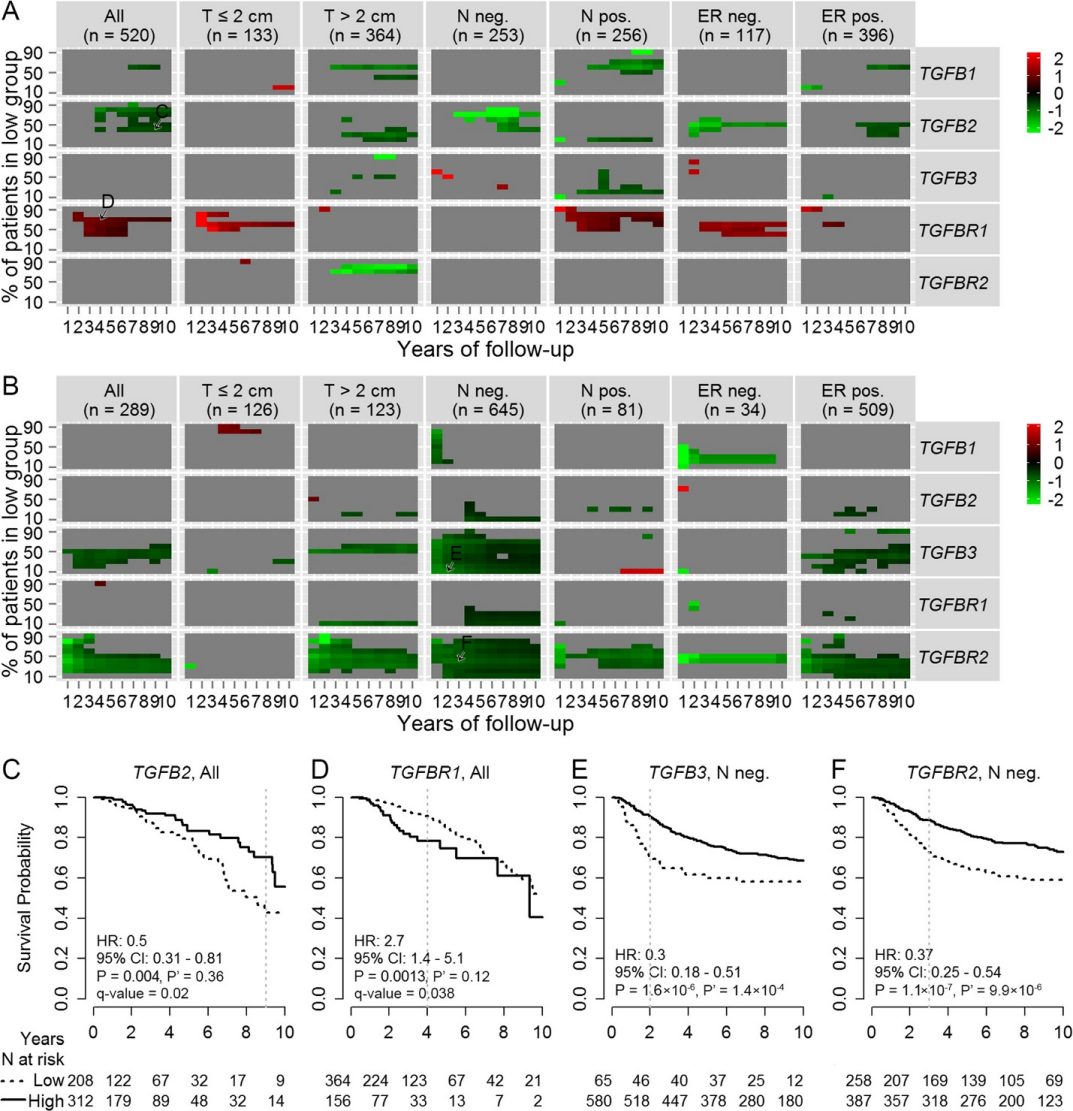
Stratifying patients by tumour mRNA levels for prognosis. Patients were divided into two groups (low and high) according to their tumour levels of *TGFB1*, *TGFB2*, *TGFB3*, *TGFBR1* or *TGFBR2* mRNA (**a**) in the TCGA cohort and its subsets and (B**b**) in the GEO cohorts. To avoid arbitrary grouping, the cut point for the percentage of patients in the group “low” was set to 10–90 % of the population with an increment of 10 % (y-axis). The hazard ratios of event (death for the TCGA cohorts and relapse for the GEO cohorts) for the patients in group “high” compared with the patients in group “low” for different periods of follow-up time (from 1 to 10 years, x-axis) were calculated using the Cox proportional hazards regression model. The corresponding Cox regression coefficients (β, β = ln(HR)) with corresponding p-values for Log-rank test <0.05 were shown as heatmap (green: low, red: high). The comparison of Kaplan-Meier estimates of survival for the arrowed data points in panels (**a**) and (**b**), which had the lowest Wald test *p*-values for HR in the corresponding block, were shown in panels (**c**), (**d**), (**e**) and (**f**) accordingly. The Kaplan-Meier survival curves were shown up to 10 years of follow-up. The statistical results shown in each plot were calculated based on different years of follow-up time as indicated by the grey dotted line. The numbers of patients at risk were listed under each time interval

Higher *TGFB2* mRNA levels in the primary tumours were associated with better overall survival of patients (Fig. 4a). The beneficial effect of having higher expression of *TGFB2* mRNA in the tumours seemed to be in the later years after diagnosis (Fig. 5a). In the patients, the hazard ratio of death for having tumours expressing the highest 60 % of *TGFB2* mRNA levels versus having tumours expressing the lowest 40 % of *TGFB2* mRNA levels for the first 9 years after diagnosis was 0.5 (95 % CI: 0.31 – 0.81, Fig. 6c). Although the p-value did not survive Bonferroni correction, it had a q-value of 0.02 representing 2 % of FDR.

Higher *TGFB3* mRNA levels in the primary tumours were associated with better relapse-free survival of patients, especially for the lymph node-negative patients and in the first 3 years after diagnosis (Figs. 4b and 5b). In patients diagnosed with lymph node-negative tumours, compared with those whose breast tumours were in the lowest 10 % of *TGFB3* expression, the other 90 % patients had significantly better relapse-free survival for the first 2 years (*P* = 1.6 × 10^−6^, P’ = 1.4 × 10^−4^) with HR = 0.3, 95 % CI: 0.18 – 0.51 (Fig. 6e).

Higher *TGFBR1* mRNA levels were associated with poorer overall survival in the first 3 years, especially for the patients with small (diameter ≤2 cm) tumours (Figs. 4a and 5a). This finding was consistent with de Kruijf et al.’s study evaluating the protein levels of TβRI by immunohistochemistry [9]. The authors also reported that high TβRII expression was associated with an unfavourable prognosis concerning progression-free survival. Whereas, it was shown in this study that higher *TGFBR2* mRNA expression was associated with better relapse-free survival for patients, especially for those with negative lymph node status and in the first 3 years (Figs. 4b, 5b and 6f). This finding was more in agreement with Paiva et al.’s study which showed that TβRII down-expression was significantly associated with breast cancer and the absence of TβRII was an adverse prognostic factor [10].

## Discussion

TGFβ signalling is well known for its involvement in a wide range of different, even opposite cellular processes, such as those involved in tumour suppression and tumour progression [4]. Tumour cells that have accumulated mutations to circumvent the suppressive effect of TGFβ may not only withstand a TGFβ-rich microenvironment but also exploit the TGFβ signalling machinery for malignant progression [1]. Thus we may assume that there are generally three types of tumour cells in terms of their responses to TGFβ: those that respond to TGFβ and are suppressed (type 1), those that do not respond to TGFβ (type 2) and those that respond to TGFβ but progress (type 3).

In breast cancer, mutations are rarely found in the TGFβ family genes [27], which implicates that the cellular context rather than the proteins in the TGFβ signalling pathway determines the action of TGFβ [4]. Thus the levels of biologically active forms of TGFβ ligands in the microenvironment and the protein expression levels of TGFβ receptors may serve as quantitative measures of the intensity of TGFβ signalling input and the responsiveness of cells to TGFβ, respectively. The protein expression levels of the TGFβ ligands may be used as the estimates of the levels of their biologically active forms, since all the TGFβ isoforms are expressed as latent forms that need to be activated to function [28]. The mRNA expression levels may be used as the estimates of the protein expression levels [29].

The *TGFBR2* mRNA levels were reduced by around two-thirds in breast tumours compared with matched normal tissues, which was consistent with a study evaluating the protein levels of TβRII [30]. The result implicated that the breast tumour cells had developed abilities to reduce their responsiveness to TGFβ by down-regulating *TGFBR2* expression. Higher *TGFB1* mRNA expression levels were observed in tumours compared with adjacent normal tissues and seemly in tumours from lymph node-positive patients compared with tumours from lymph node-negative patients. It implicated that the malignant tumour cells up-regulated *TGFB1* expression. The *TGFB3* mRNA expression levels were around 50 % lower in the ER-negative/PR-negative/triple-negative/Basal-like/Grade 3 tumours, which were all associated with poor prognosis. As *TGFB3* mRNA levels were increased by 84 % in tumours compared with adjacent normal tissues, the results should be interpreted as *TGFB3* expression was greatly up-regulated in premalignant tumour cells and/or their surrounding cells (the bio-specimens were usually composed of <100 % tumour cells), rather than *TGFB3* expression was down-regulated in malignant tumour cells. The up-regulated *TGFB1* expression in malignant tumour cells and the up-regulated *TGFB3* expression in premalignant tumour cells were very likely to have different, even opposite effects on the corresponding tumours, which also implicated the specific biology of different TGFβ isoforms.

Assuming that we can group breast cancer patients according to the tumour cells’ responses to TGFβ, and evaluate the associations between the expression levels of *TGFB1*, *TGFB2*, *TGFB3*, *TGFBR1* and *TGFBR2* and patients’ clinical outcomes such as overall and relapse-free survival in each group using Cox proportional hazards regression model, then we should expect negative log hazard ratios for type 1 tumours, log HRs of zero for type 2 tumours and positive log HRs for type 3 tumours, respectively. Obviously, the HRs for different genes should be different. Unfortunately, we are still not able to separate the three types of tumours. However, for a cohort composed of patients with mixed types of tumours, a significant negative log HR implicates that there are more patients with type 1 tumours, a significant positive log HR implicates that there are more patients with type 3 tumours, and a non-significant HR implicates that there are more patients with type 2 tumours, the patients are heterogeneous or the population is too small. Thus the association between the expression levels and patients’ clinical outcomes may serve as an indicator of the proportion of the three types of tumours. The conflicting results such as that were reported for the associations between the protein levels of TGFβ1 and TβRII and the patients’ clinical outcomes [8–11], were very likely due to that the studied cohorts were composed of different proportions of the three types of tumours.

The significant positive associations observed between tumour levels of *TGFB2*, *TGFB3* and *TGFBR2* mRNA and clinical outcomes in patients diagnosed with lymph node-negative diseases implicated that the majority of the tumours from those patients still responded to the suppressive effect of TGFβ signalling, and the tumours with lower *TGFB2*, *TGFB3* and *TGFBR2* expression were more advantageous in tumour progression. In patients diagnosed with small tumours, those with higher *TGFBR1* mRNA levels in the tumours had much poorer prognosis for the first 3 years. One of the rationales for anti-cancer drugs targeting the TGFβ pathway is to block the TGFβ signalling for type 3 tumours but not type 1 or type 2 tumours. Our data implicated that in the patients with small breast tumours, there was a high proportion of patients with type 3 tumours that might be more likely to benefit from the drugs targeting TβRI.

## Conclusion

In conclusion, the mRNA levels of the TGFβ isoforms and receptors in breast tumours were differentially associated with patients’ overall and relapse-free survival in patients stratified by different tumour characteristics. Before we can develop techniques to precisely classify breast tumours into different types according to their responses to TGFβ, the associations between the tumour mRNA levels and patients’ clinical outcomes may not only provide prognostic value for patients but also assist in classifying tumours according to their potential responses to TGFβ and selecting patients for the TGFβ signalling pathway targeted therapies that are under development [6]. The mechanisms underlying the reduced *TGFBR2* expression in tumours and the differentially regulated *TGFB1* and *TGFB3* expression by malignant and premalignant tumour cells may also have potential clinical implications that need to be further explored.

## References

[1] Massague J (2008). TGFbeta in cancer. Cell.

[2] Pickup M, Novitskiy S, Moses HL (2013). The roles of TGFbeta in the tumour microenvironment. Nat Rev Cancer.

[3] Shi Y, Massague J (2003). Mechanisms of TGF-beta signaling from cell membrane to the nucleus. Cell.

[4] Massague J (2012). TGFbeta signalling in context. Nat Rev Mol Cell Biol.

[5] Zu X, Zhang Q, Cao R, Liu J, Zhong J, Wen G (2012). Transforming growth factor-beta signaling in tumor initiation, progression and therapy in breast cancer: an update. Cell Tissue Res.

[6] Akhurst RJ, Hata A (2012). Targeting the TGFbeta signalling pathway in disease. Nat Rev Drug Discov.

[7] Baxley SE, Serra R (2010). Inhibiting breast cancer progression by exploiting TGFbeta signaling. Curr Drug Targets.

[8] Grau AM, Wen W, Ramroopsingh DS, Gao YT, Zi J, Cai Q (2008). Circulating transforming growth factor-beta-1 and breast cancer prognosis: results from the Shanghai Breast Cancer Study. Breast Cancer Res Treat.

[9] de Kruijf EM, Dekker TJ, Hawinkels LJ, Putter H, Smit VT, Kroep JR (2013). The prognostic role of TGF-beta signaling pathway in breast cancer patients. Ann Oncol.

[10] Paiva CE, Drigo SA, Rosa FE, Moraes Neto FA, Caldeira JR, Soares FA (2010). Absence of transforming growth factor-beta type II receptor is associated with poorer prognosis in HER2-negative breast tumours. Ann Oncol.

[11] Ciftci R, Tas F, Yasasever CT, Aksit E, Karabulut S, Sen F (2014). High serum transforming growth factor beta 1 (TGFB1) level predicts better survival in breast cancer. Tumour Biol.

[12] Amoils KD, Bezwoda WR (1997). TGF-beta 1 mRNA expression in clinical breast cancer and its relationship to ER mRNA expression. Breast Cancer Res Treat.

[13] MacCallum J, Bartlett JM, Thompson AM, Keen JC, Dixon JM, Miller WR (1994). Expression of transforming growth factor beta mRNA isoforms in human breast cancer. Br J Cancer.

[14] Cancer Genome Atlas N (2012). Comprehensive molecular portraits of human breast tumours. Nature.

[15] Parker JS, Mullins M, Cheang MC, Leung S, Voduc D, Vickery T (2009). Supervised risk predictor of breast cancer based on intrinsic subtypes. J Clin Oncol.

[16] Ivshina AV, George J, Senko O, Mow B, Putti TC, Smeds J (2006). Genetic reclassification of histologic grade delineates new clinical subtypes of breast cancer. Cancer Res.

[17] Schmidt M, Bohm D, von Torne C, Steiner E, Puhl A, Pilch H (2008). The humoral immune system has a key prognostic impact in node-negative breast cancer. Cancer Res.

[18] Wang Y, Klijn JG, Zhang Y, Sieuwerts AM, Look MP, Yang F (2005). Gene-expression profiles to predict distant metastasis of lymph-node-negative primary breast cancer. Lancet.

[19] Symmans WF, Hatzis C, Sotiriou C, Andre F, Peintinger F, Regitnig P (2010). Genomic index of sensitivity to endocrine therapy for breast cancer. J Clin Oncol.

[20] Yuan JS, Reed A, Chen F, Stewart CN (2006). Statistical analysis of real-time PCR data. BMC Bioinformatics.

[21] Vandesompele J, De Preter K, Pattyn F, Poppe B, Van Roy N, De Paepe A (2002). Accurate normalization of real-time quantitative RT-PCR data by geometric averaging of multiple internal control genes. Genome Biol.

[22] Kleinbaum DG, Klein M. Extension of the cox proportional hazards model for time-dependent variables. Stat Biol Health. 2012:241–288. DOI 10.1007/978-1-4419-6646-9_6

[23] Storey JD (2002). A direct approach to false discovery rates. J R Stat Soc B.

[24] De Crescenzo G, Hinck CS, Shu Z, Zuniga J, Yang J, Tang Y (2006). Three key residues underlie the differential affinity of the TGFbeta isoforms for the TGFbeta type II receptor. J Mol Biol.

[25] Laverty HG, Wakefield LM, Occleston NL, O’Kane S, Ferguson MW (2009). TGF-beta3 and cancer: a review. Cytokine Growth Factor Rev.

[26] Figueroa JD, Flanders KC, Garcia-Closas M, Anderson WF, Yang XR, Matsuno RK (2010). Expression of TGF-beta signaling factors in invasive breast cancers: relationships with age at diagnosis and tumor characteristics. Breast Cancer Res Treat.

[27] Kandoth C, McLellan MD, Vandin F, Ye K, Niu B, Lu C (2013). Mutational landscape and significance across 12 major cancer types. Nature.

[28] Annes JP, Munger JS, Rifkin DB (2003). Making sense of latent TGFbeta activation. J Cell Sci.

[29] Vogel C, Marcotte EM (2012). Insights into the regulation of protein abundance from proteomic and transcriptomic analyses. Nat Rev Genet.

[30] Gobbi H, Arteaga CL, Jensen RA, Simpson JF, Dupont WD, Olson SJ (2000). Loss of expression of transforming growth factor beta type II receptor correlates with high tumour grade in human breast in-situ and invasive carcinomas. Histopathology.

